# Supplementary material for: Association between oral microbiome and seven types of cancers in East Asian population: a two-sample Mendelian randomization analysis
Source: Front Mol Biosci. 2023 Nov 21;10:1327893. doi: 10.3389/fmolb.2023.1327893 (PMC10702768; doi:10.3389/fmolb.2023.1327893)
Supplement: Supplementary file 1 [file DataSheet1.ZIP › Supplementary Materials/MR plots of saliva/saliva═╝/Lung Cancer/pheno.2509_to_lung cancer_leave_one_out.pdf]

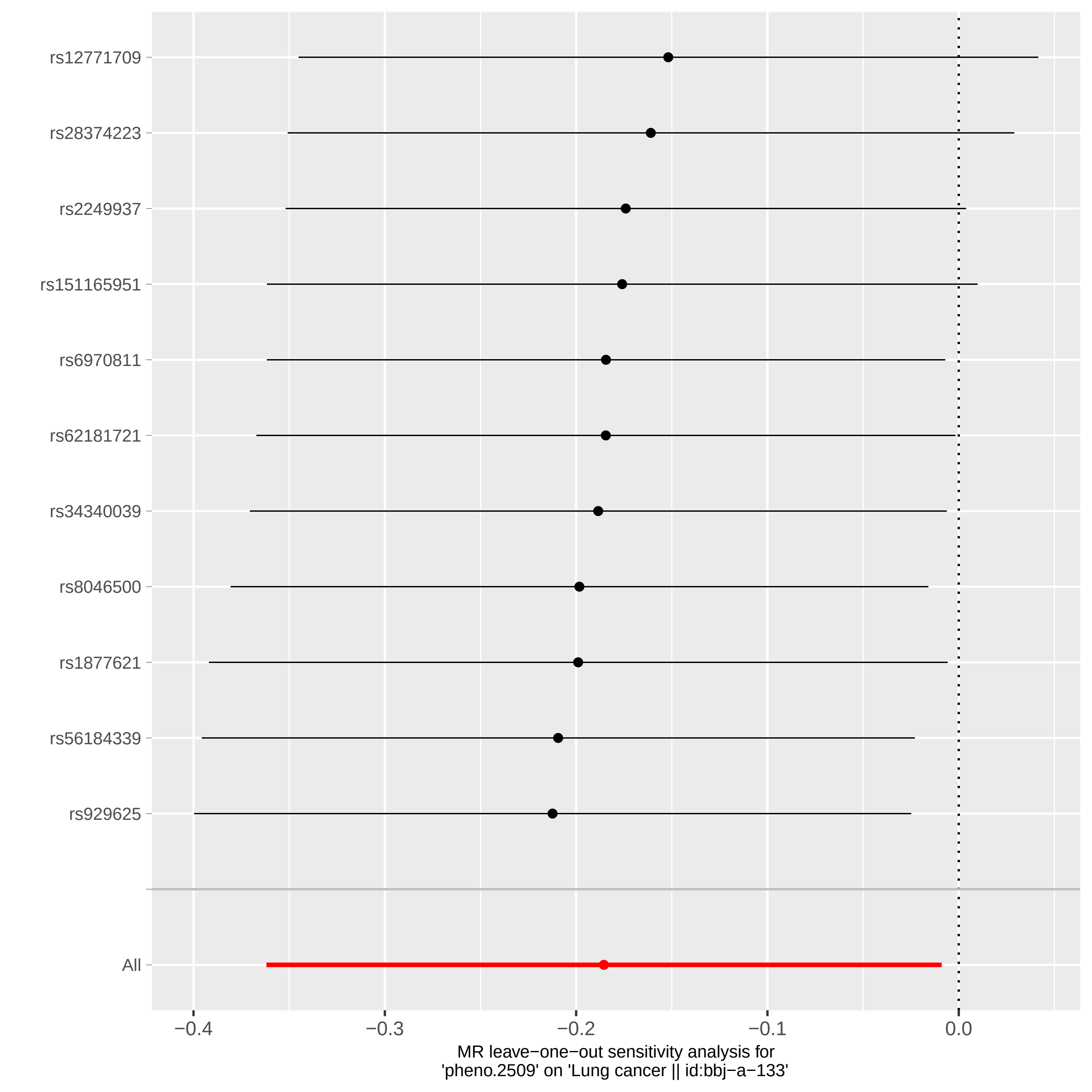
rs12771709
rs28374223
rs2249937
rs151165951
rs6970811
rs62181721
rs34340039
rs8046500
rs1877621
rs56184339
rs929625
All
−0.4
−0.3
−0.2
−0.1
0.0
MR leave−one−out sensitivity analysis for
'pheno.2509' on 'Lung cancer || id:bbj−a−133'
